# Particle simulation approach for subcellular dynamics and interactions of biological molecules

**DOI:** 10.1186/1471-2105-7-S4-S20

**Published:** 2006-12-12

**Authors:** Ryuzo Azuma, Tetsuji Kitagawa, Hiroshi Kobayashi, Akihiko Konagaya

**Affiliations:** 1 RIKEN Genomic Sciences Center, 1-7-22 Suehiro Tsurumi, Yokohama, Kanagawa, 230-0045, Japan; 2 Dept. of Mathematics and Computing Sciences, Tokyo Institute of Technology, 2-12-1 Ookayama, Meguro, Tokyo, 152-8552, Japan; 3 Graduate School of Pharmaceutical Sciences, Chiba University, 1-8-1 Inohana, Chuo, Chiba, 260-8675, Japan

## Abstract

**Background:**

Spatio-temporal dynamics within cells can now be visualized at appropriate resolution, due to the advances in molecular imaging technologies. Even single-particle tracking (SPT) and single fluorophore video imaging (SFVI) are now being applied to observation of molecular-level dynamics. However, little is known concerning how molecular-level dynamics affect properties at the cellular level.

**Results:**

We propose an algorithm designed for three-dimensional simulation of the reaction-diffusion dynamics of molecules, based on a particle model. Chemical reactions proceed through the interactions of particles in space, with activation energies determining the rates of these chemical reactions at each interaction. This energy-based model can include the cellular membrane, membranes of other organelles, and cytoskeleton. The simulation algorithm was tested for a reversible enzyme reaction model and its validity was confirmed. Snapshot images taken from simulated molecular interactions on the cell-surface revealed clustering domains (size ~0.2 μm) associated with rafts. Sample trajectories of raft constructs exhibited "hop diffusion". These domains corralled the diffusive motion of membrane proteins.

**Conclusion:**

These findings demonstrate that our approach is promising for modelling the localization properties of biological phenomena.

## Background

We propose here a general method of simulating the dynamics and interactions of molecules based on a particle framework. Analyses of subcellular localization are now quite important to know how the cellular properties of interest are regulated. Experimental techniques helped unraveling these properties. For example, single particle tracking (SPT) and single fluorophore video imaging (SFVI) techniques have enabled observation of how individual molecules actually move and interact in space and time. SPT and SFVI have been used to investigate the dynamics of receptors in the plasma membrane [1,2] and mRNAs in the nucleus [3-5]. These techniques have also enabled the sizes of microdomain structures [6,7] to be measured. While some of these observations provided quantitative data, as in the case of SPT/SFVI studies, most merely provided a multitude of qualitative information focused primarily on biological significance. Moreover, the length and time scales in these experiments were approximately intermediate in scale between those that may be addressed by typical microscopic and macroscopic simulations (i.e., molecular dynamics and rate equations). Hence, our objective was to provide a simulation tool useful for integrating and examining experimental data quantitatively at the "mesoscopic" scale.

Our simulation method incorporates Brownian motion of molecules in 3D space. Interactions of molecules in the space are due to the production of complexes. The association and dissociation of these complexes, coupled with the modification of substrates to products, are accepted at certain rates based on a Monte Carlo (MC) algorithm which takes account of changes in their energy states. Although our setup is based on this type of molecular-level description, its ensemble average has been confirmed to correctly reproduce predictions derived from thermodynamic and rate equation theories. By using this method we succeeded to demonstrate the production of clustering patterns on the cellular membrane associated with cholesterol-rich detergent-resistant membranes (DRM) termed "rafts" [8]. Furthermore, there we obtained important observations implying (1) molecular trajectories of raft constructs give rise to characteristic types of diffusion associated with "hop diffusion" [9] (2) the production of membrane protein complexes are facilitated by entering a clustering domain (3) the escaping rate of protein complexes from the clustering domain seems to be less than that of unbound molecules (4) hence the membrane proteins are corralled in these domains most of the time, and in turn appear to stabilize the clustering domains. These results suggest the usefulness of our simulation approach.

This paper is organized as follows. In **Methods **we explain the random walk, binding, dissociation, and catalysis processes in our MC simulation algorithm. Then we discuss the correspondence of our probability constants to kinetic parameters of the corresponding rate equations. In **Results **we compare our simulation result of a reversible enzyme reaction model with the prediction from the rate equation theory. Then we show the result of cell-surface clustering simulation. The final section is devoted to our conclusions.

## Methods

### The random walk process

In this process, each particle moves along a cubic lattice, taking random steps to reach one of the six nearest neighbor sites with equal probability. The steps are of length *l*, so that each subsequent particle position can only take the values (*n*_*x*_*l*, *n*_*y*_*l*, *n*_*z*_*l*), where *n*_*x*_, *n*_*y*_, and *n*_*z *_are integers. This process allows the particle to take steps with probability *d *per unit time (τ), meaning that the particle waits at each point for a variable amount of time. A master equation theory can show that at the limit *l *→ 0, this type of random walk can be considered a Wiener process with a time-dependent Gaussian distribution with the following diffusion coefficient [10]:



The fastest rate of diffusion here is equivalent to *d *= 1/6[τ^-1^], meaning that the particle must take the random walk step.

### The binding process

**Figure 1:**
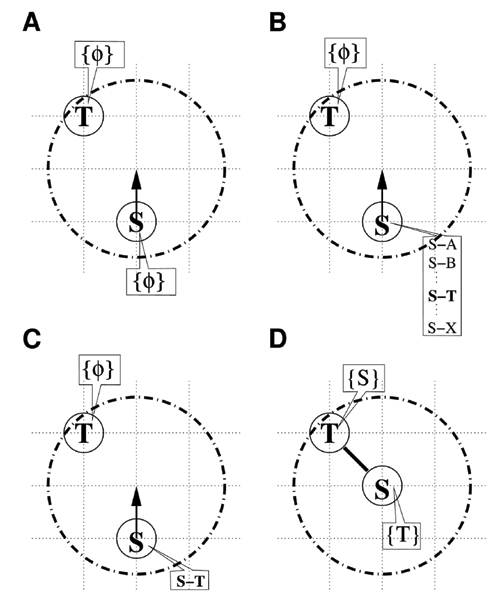
**The binding process. **(A) The movement trial drives S upward. Here the bond variables of S and T are empty (symbols {φ}). (B) Before the movement, S is checked to determine whether it may bind with T, by searching among the complex candidates. (C) If the table has a combination of single S and single T (S-T) simultaneously, a uniform random number ξ (0 ≤ ξ < 1) and the transition probability *p*_1 _are compared. (D) When ξ <*p*_1_, the movement is accepted.

Suppose that a particle of chemical species S has just entered the interaction range of another particle of T species through the movement trial described above, and that these particles may bind to each other. In this binding process, whether the particles can form a ST complex must be determined. Specifically, we need to obtain both the candidates for complexes in a predefined table and the complexes that have already formed. Figure 1 shows a typical example of the case of a binary complex. For simplicity, we consider only the dynamics projected onto a particular plane. Here, S, T, and U denote the chemical species of particles. The area enclosed by the dash-dot circle indicates the interaction range for particle S, which is defined as a sphere of radius √3. In this case, the binding process consists of the series of steps listed below.

1. Particle S moves upward, as indicated by the pointer, causing another particle T to enter the interaction range. Here, the symbols {φ} attached to these particles denote an empty variable and indicate the absence of bound particles. The variable is hereinafter referred to as the bond variable (Figure 1A).

2. The ability of S to bind with T is checked by referring to the predefined table for S. More precisely, when the table has a combination of single S and single T (represented by S-T in (b)), particle S is able to bind with particle T (Figure 1B).

3. A uniform random number ξ (0 ≤ ξ < 1) is generated and compared with probability *P*(ST|S+T) = exp(-Δ*E*_1_/*k*_B_*T*) ≡ *p*_1_, where Δ*E*_1_/*k*_B_*T *is a dimensionless activation energy. When ξ <*p*_1_, the movement of particle S is accepted; when ξ ≥ *p*_1_, it is rejected (Figure 1C). Here we assume that the rate of the production of ST complex is related to the conditional probability *p*_1_.

4. When the movement is accepted, we assign T to the bond variable of particle S, and vice versa (Figure 1D).

### Conservation of stoichiometry

**Figure 2:**
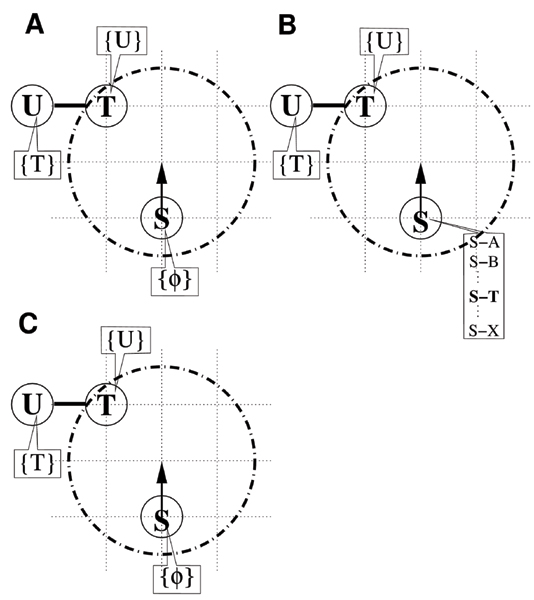
**An interaction without binding. **(A) U already occupies the bond variable of T due to the binding of T and U. (B) The process of searching for TS among the candidates. (C) Particle T returns {U}, with discarding of the ST complex.

It is not necessarily true that all pairs of S and T particles within the interaction range can bind to each other. Figure 2 shows this exceptional case, in which molecule T has already bound to a molecule of species U and therefore cannot bind to molecule S. In this case, these particles undergo the series of steps listed below.

1. The movement trial drives particle S upward, thereby causing particle T to enter the interaction range (Figure 2A).

2. By referring to the predefined table, it is found that molecule S can bind with molecule T (Figure 2B).

3. The bond variable in molecule T is checked, yielding species U. This prevents the assignment of T to the bond variable of particle S, since S cannot be assigned to the variable of T (Figure 2C).

### The dissociation process

In this process, the species S and T in the bond variables are assigned to particles T and S, respectively, and are cleared upon the acceptance of unbinding.

**Figure 3:**
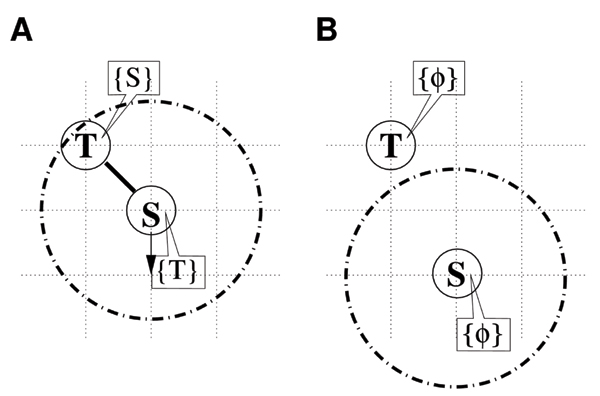
**The unbinding process. **Particle S is driven downward by the random walk trial. The TS complex (A) splits into the S and T particles (B).

1. A uniform random number ξ (0 ≤ ξ < 1) is generated and compared with probability *P*(S+T|ST) = exp(-Δ*E*_2_/*k*_B_*T*) ≡ *p*_2_, where Δ*E*_2_/*k*_B_*T *is a dimensionless activation energy. When ξ <*p*_2_, the movement of particle S is accepted; when ξ ≥ *p*_2_, it is rejected (Figure 3A). The basis of this mechanism is Kramers' theory, which predicts that the reaction rate of S+T→ST can be written as an exponential function of Δ*E*_2_/*k*_B_*T *[11].

2. When the movement is accepted, T in the bond variable of particle S is cleared, and vice versa (Figure 3B).

### The modification process

**Figure 4:**
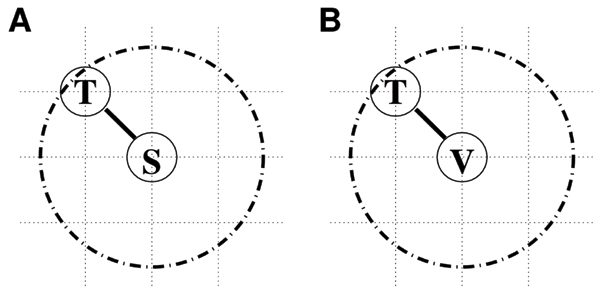
**The modification process. **The TS complex (A) is converted to TV (B).

This process allows each particle to undergo a trial in which its chemical species is replaced by a different one. The steps of the procedure are as follows:

1. A uniform random number ξ (0 ≤ ξ < 1) is generated and compared with probability *P*(VT|ST) = exp(-Δ*E*_3_/*k*_B_*T*) ≡ *p*_3_, where Δ*E*_3_/*k*_B_*T *is a dimensionless activation energy. When ξ <*p*_3_, the modification of particle S to V is accepted; when ξ ≥ *p*_3_, it is rejected (Figure 4A).

2. When the modification is accepted, S is replaced by V (Figure 4B).

### Time and length scales

We define unit time τ of a simulation as a cycle in which every particle has undergone the movement trial only once. Also the trial of the modification process is performed only once for every particle per single unit time. We then relate the unit time and unit length to real ones. Specifically, our choices of scale conversions are: (a) when we are interested in relatively fast dynamics within a small volume, 1 sec ≡ 5 × 10^5^τ and 1 μm ≡ 181.9*l*, or (b) when we study the long-time behavior of reactions within a relatively large volume, 1 sec ≡ 5 × 10^3^τ and 1 μm ≡ 18.19*l *(Table 1).

These two combinations of scale conversions are selected so that both give *D *≈ 7.6 [μm^2^/sec] when *d *= 1/6[τ^-1^], i.e, the fastest diffusion. This is readily checked by the following calculation. Since the diffusion coefficient is estimated using *D *≡ 3*l*^2^*d*[τ^-1^] as an approximation of Equation (1), and by noting that *l *= 5.498 × 10^-3 ^[μm] and *d *= 5 × 10^5^/6 [sec^-1^] for case (a), and *l *= 5.498 × 10^-2 ^[μm] and *d *= 5 × 10^3^/6 [sec^-1^] for case (b), we obtain *D *= 3*l*^2^*d*[τ^-1^] ≈ 7.6 [μm^2^/sec] equally for (a) and (b). This value approximates experimental values for globular proteins in the cytoplasm [12].

### Kinetic coefficients

Table 1 summarizes the correspondence between the probability constants and kinetic coefficients theoretically derived for cases (a) and (b). Here, *k*_1_, *k*_-1_, and *k*_2 _denote the kinetic coefficients for reactions S+T→ST, ST→S+T, and ST→VT, respectively. The second-order kinetic coefficient (*k*_1_) is approximated by 4π*R *× 2*D *× *p*_1_, as predicted from diffusion-limited reaction rate theory in three-dimensional solutions in the limit case of *t *→ ∞ [13,14]. Here, *R *is the reaction radius and approximated by *l*. The factor 1/3 in *k*_-1 _indicates that the total number of movements with unbinding for all possible binding conformations of S for fixed T is one-third of the total number of movements, with or without unbinding for all possible binding conformations of S for fixed T.

## Results

### Dynamics of bound molecules

**Figure 5:**
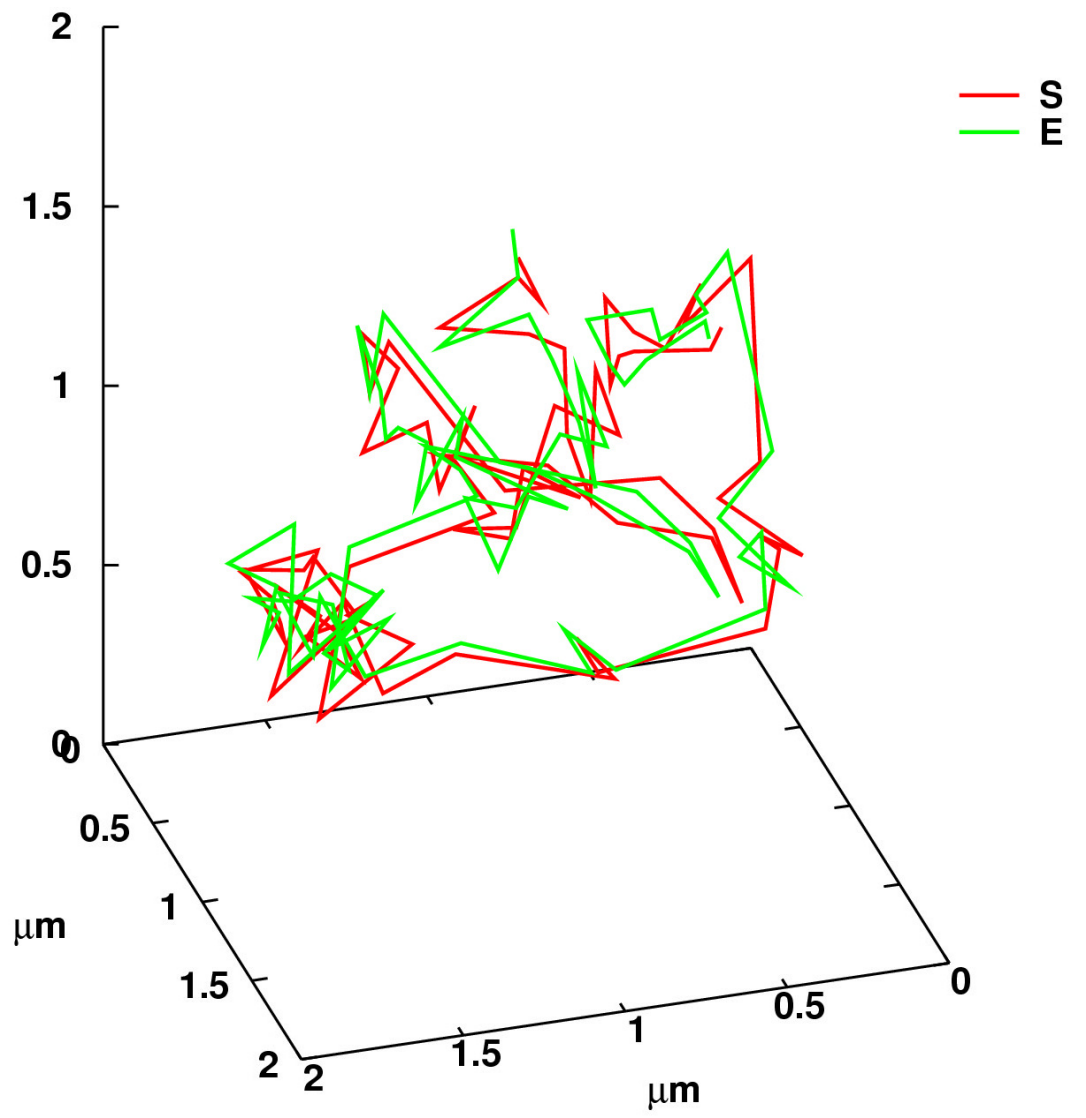
**S+E->SE particle simulation. **Trajectories of particles S and E bind together with *p*_2 _= 0, taken at every 1.6 × 10^2^τ from *t *= 0 to 1.6 × 10^4^τ. The unit of length is 1 μm, based on the scale conversion of (b) in Table I. Red: particle of S species; Green: particle of E species.

To show that our algorithm and its implementation – especially for the binding process – correctly function as designed in a simulation, Figure 5 displays a trajectory of an SE complex with *p*_2 _= 0 (i.e., permanent binding). Within this time range, molecules S and E remain continually bound to each other, while undergoing random movements in space (a movie presentation of this can be obtained in [15]).

### Reversible enzyme reaction

To determine in more quantitative fashion whether our technique satisfies the theoretical requirements for the binding, unbinding, and modification processes, we conducted a series of simulations for the following reversible enzyme reactions:



Then we compared these reactions with the results obtained from rate equations. Here, the variables in each reaction denote the combination of a kinetic coefficient and dimensionless activation energy (e.g., *a*_1 _and ΔEf1 in S+E→SE reaction).

**Figure 6:**
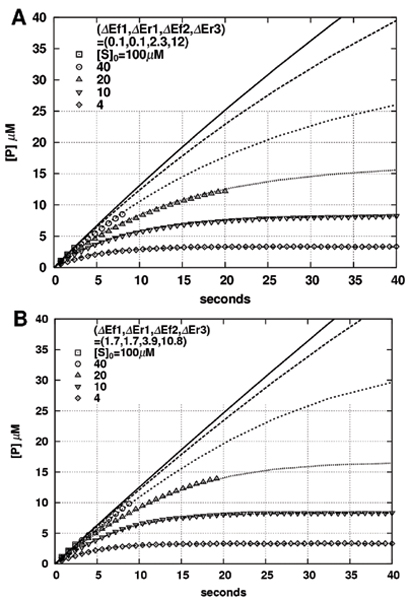
**Comparison of particle simulation and ODE results. **The average of [P] plotted with time (sec) for Monte Carlo (particle) simulations (open symbols) and the corresponding rate equations (line curves). The activation energies and their corresponding kinetic parameters are listed in Table 2: (a) and (c) in Table 2 for (A), while (b) and (d) in Table 2 for (B). The total concentration of E is [E]_0 _= 0.1 μM. The initial distribution is random with equal probability for all the sites in the 3D space, with use of 16 samples.

In our Monte Carlo (MC) simulations, we estimated average time evolutions over 16 samples, starting with a random initial distribution of particles. Figure 6 plots the average of time evolution of [P] for various initial concentrations [S]_0 _and two sets of activation energies listed in Table 2(a) and 2(b) (open symbols in Figure 6A and 6B, respectively). We confirmed that the ratio [P]/[S] of steady-state values correctly reproduces exp(-ΔG/*k*_B_*T*) (e.g., coincidence between steady state values for [S]_0 _= 10 (or 4) with plotting symbol ▽ (or ◊) in Figure 6A and 6B), where ΔG = ΔEf1-ΔEr1-ΔEf2+ΔEr2+ΔEf3-ΔEr3 = 0.7*k*_B_*T*.

To make comparisons with rate equations, we applied the scale conversion rule in Table 1(a) to the activation energies in Table 2(a) and 2(b), thereby obtained the corresponding kinetic parameters in Table 2(c) and 2(d), respectively. The curved lines in Figure 6 are then drawn by numerically solving the following rate equations:



with the following constraints:



where, *x*, *y*, and *z *denote [SE], [PE], and [P], respectively.

### Extension to higher-order chemical reactions

The simulations thus far described have included only chemical reactions with complexes consisting of at most two different species. For general application of our simulation method, however, we must incorporate algorithms that address higher-order chemical reaction models that involve many-body interactions with more than two species. To do this, we developed an extended version of the algorithm by incorporating "bond tables" in which the indexes and species of bound partners for each particle are continually updated. We will not describe further details of the algorithm here because of their complexity, and will note only that they conform in essence to what we have described in **Methods**.

### Clustering on the cellular membrane

The advantages of this type of strict stoichiometric processing of interactions are enormous. One of the best demonstrations of the usefulness of the extended algorithm is simulation of clustering on the cellular membrane. This simulation examines the effects of associating LAT, a transmembrane adaptor protein, with T-cell receptor TCR on the lifetime of raft clustering. The T-cell receptor and LAT are shown to preferentially associate with cholesterol-rich microdomains on the cell surface.

Cholesterol has very important effects on the clustering, since it can change the properties of plasma membranes that mainly consist of sphingomyelins. The addition of cholesterol is shown to drive a solid-ordered (SO) phase transition into a liquid-ordered (LO) one. In the intermediate level of this addition, the LO phase and liquid-disordered (LD) phase may coexist. This may give rise to lateral heterogeneity in the plasma membrane, thereby segregating cholesterols into cholesterol-rich domains [8]. The effects of other factors relevant to clustering in the outer leaflet of plasma membranes are also emphasized. It has been proposed that the length and saturation of alkyl chains are responsible for clustering: glycosphingolipids, sphingomyelins, and phospholipids with long saturated alkyl chains may constitute rafts by entering the cholesterol-rich domains [16-19].

**Figure 7:**
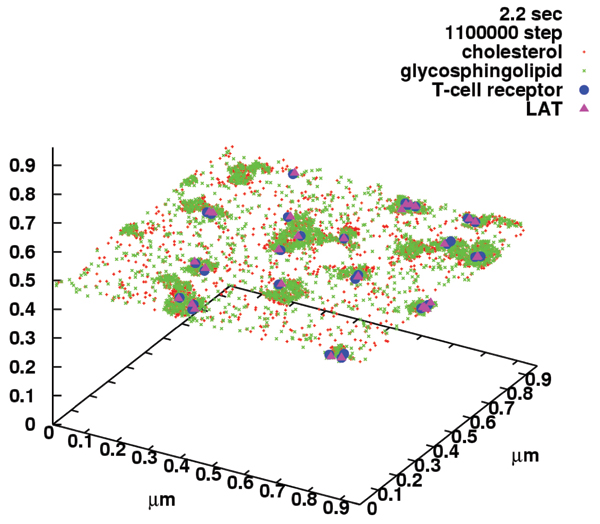
**Simulation of cell-surface clustering. **A snapshot image taken at t = 2.2 sec. The length scale of each side is in μm. Molecular species: (+) cholesterol, (×) glycosphingolipids, (●) T-cell receptor, and (▲) LAT adaptor protein.

Taking these factors into account, we incorporated key components and their mutual interaction parameters other than those related to constituents in the bulk domain. We considered the interactions among cholesterols (component C), glycosphingolipids (component G), TCR (component T), and LAT (component L) molecules: a lower magnitude of coupling is assumed for CG, GT, and GL complexes, and a higher affinity is assumed for the TL complex (for details see [20]). With this setup and a sufficient amount of CG complexes, stable clustering patterns are obtained (Figure 7; movie file is in [21]). In Figure 7C and 7G, components segregate and form clustering domains (size ~0.2 μm), reproducing DRM/raft-like structures. The improvement made in our simulation from those described in previous studies [18] is demonstration of oligomerization-induced clustering, whereby the presence of a small number of plasma membrane protein complexes bound with relatively strong coupling correlates with the production of stabilized rafts (receptor-cluster rafts) that are rich in C and G [22]. In fact, the production of TL complexes is facilitated by the corralling of T and L in the clustering domains, while the slow motion of the TL complexes within the clusters in turn appears to hinder breakup of the clusters.

**Figure 8:**
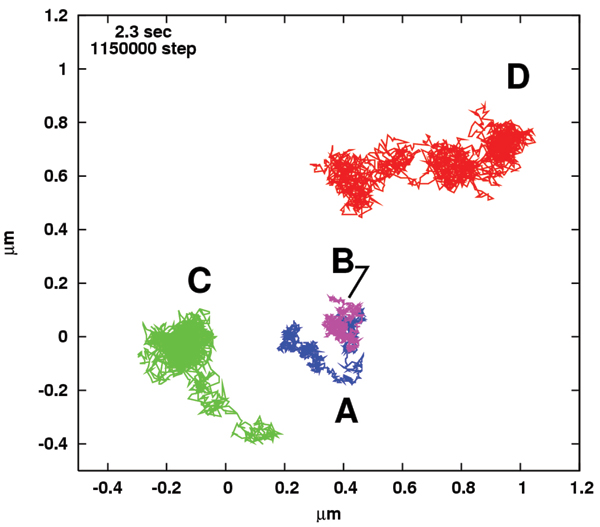
**Simulation of cell-surface clustering: Particle trajectories. **(A) TCR, (B) LAT, (C) cholesterol, and (D) glycosphingolipid, for t = 0.4–2.3 sec.

To make comparisons with SPT as well as SFVI experiments, in particular, we displayed typical sample trajectories obtained from this simulation (Figure 8, movie file is in [23]). We observed two types of diffusional movements in the presence of clustering: the slow movement observed mainly for T and L (Figure 8A and 8B), and the relatively free motion exhibited by C and G (Figure 8C and 8D) but with a slower diffusion rate than that in the absence of clustering. Interestingly, these trajectories, especially that of G in Figure 8D, appear to exhibit lumps with a similar size (~0.2 μm), indicating "hop diffusion" across clusters. This size approximately equals the diameter of the clustering domain in Figure 7. For T and L, this hop diffusion rarely occurs, because most T's and L's rapidly form TL complexes in the clusters, and thus the energy cost of escaping from them increases. Hence, in Figures 8A and 8B, T manages to escape from a cluster and enters another one wherein L remains to be trapped. Experimentally, images of trajectories of single or small groups of DOPE (a phospholipid) molecules on the membrane of an NRK fibroblastic cell recorded at time resolutions of 25 μs were tracked, and found to exhibit hop diffusion with a typical scale of around 0.2 μm [9]. In the dynamics of membrane proteins, the rate of diffusion of Lck (a Src family kinase recruited to TCR clustering) was reduced with decreasing distance from the point of stimulation in a TCR cluster [24]. These observations are consistent with our simulation results.

## Conclusions

Our simulation method represents molecular movements and interactions with the use of a particle model. The movements are expressed as a random walk in 3D space, and interactions are expressed as inter-particle binding, unbinding, and modification processes. A characteristic feature of our approach is that it enables complexes to be modeled as bound particles. The binding, unbinding, and modification processes of these complexes are based on energetic considerations, in which these processes proceed according to transition probabilities determined by the activation energy. This does not require setup of sub-volumes assuming well-stirred surrounding media, as incorporated in previous reaction-diffusion methods [25,26] based on Gillespie's algorithm [27,28]. Instead, our method requires precise treatment of the stoichiometry of these processes.

Moreover, it is important to examine the magnitude and effects of stochastic fluctuations, since our particle simulation model is essentially probabilistic. Our quantification of simulation results was intended to demonstrate that it correctly reproduced theoretical curves from a rate equation theory for the reversible enzyme reaction model. However, by observing raw time courses obtained for individual samples, continual fluctuations can be observed in raw time courses around the average value. This type of fluctuation corresponds to intrinsic noise, whereas fluctuation due to external input is referred to as extrinsic noise [29,30]. We are now performing systematic analyses of quantification of intrinsic noise by examining its dependence on kinetic constants such as *K*_m _and *V*_max_.

Using this simulation method, we successfully demonstrated the existence of a clustering pattern that may be associated with receptor-cluster rafts and the "fluid mosaic model" [31,32] in the plasma membrane. In immune cell signalling, rafts are hypothesized to be "platforms" for raft-philic adaptor proteins such as LAT in T-cells [33,34], with these proteins insulated from others with different signals. Using simulation to verify this will require (1) automatic identification of clustering domains or rafts, and (2) analyses of the net flow transferred between these domains. These topics will be addressed in the near future.

## Figures and Tables

**Table 1:** Relationships between probability constants and kinetic coefficients.

	(a)	(b)
1 sec	5 × 10^5^τ	5 × 10^3^τ
1 μm	181.9*l*	18.19*l*
*D *[μm^2^/sec]	45*d*	45*d*
*k*_1 _[nM^-1^sec^-1^]	38*p*_1_*d*	38*p*_1_*d*
*k*_-1 _[sec^-1^]	5 × 10^5 ^× 1/3 × *p*_2_*d*	5 × 10^3 ^× 1/3 × *p*_2_*d*
*k*_2 _[sec^-1^]	5 × 10^5^*p*_3_	5 × 10^3^*p*_3_

Scale conversions for cases (a) and (b), and correspondence between kinetic coefficients and probability constants.

**Table 2:** Parameter values of the reversible enzyme reaction model in Eq. 2. The translation of activation energies into kinetic coefficients are performed based on Table 1(a). *K*_eq _= *a*_1_*a*_2_*b*_-1_/*a*_-1_*b*_2_*b*_1_

Activation energy [*k*_B_*T*]	(a) (Figure 6A)	(b) (Figure 6B)	Kinetic coefficients	(c) (Figure 6A)	(d) (Figure 6B)
ΔEf1	0.1	1.7	*a*_1 _[nM^-1^sec^-1^]	5.73	1.16
ΔEr1	0.1	1.7	*b*_1 _[nM^-1^sec^-1^]	5.73	1.16
ΔEf2	2.3	3.9	*a*_-1 _[sec^-1^]	2.79 × 10^3^	5.62 × 10^2^
ΔEr2	4.6	4.6	*b*_-1 _[sec^-1^]	2.79 × 10^2^	2.79 × 10^2^
ΔEf3	10.8	10.8	*a*_2 _[sec^-1^]	1.02 × 10^1^	1.02 × 10^1^
ΔEr3	12.4	10.8	*b*_2 _[sec^-1^]	2.06	1.02 × 10^1^
ΔG	0.7	0.7	*K*_eq_	0.50	0.50
